# Retrospective Analysis Shows That Most RHDV GI.1 Strains Circulating Since the Late 1990s in France and Sweden Were Recombinant GI.3P–GI.1d Strains

**DOI:** 10.3390/genes11080910

**Published:** 2020-08-09

**Authors:** Joana Abrantes, Ana M. Lopes, Evelyne Lemaitre, Harri Ahola, Fereshteh Banihashem, Clément Droillard, Stéphane Marchandeau, Pedro J. Esteves, Aleksija Neimanis, Ghislaine Le Gall-Reculé

**Affiliations:** 1 CIBIO/InBio-UP, Centro de Investigação em Biodiversidade e Recursos Genéticos, Universidade do Porto, 4485-661 Vairão, Portugal; jabrantes@cibio.up.pt (J.A.); analopes@cibio.up.pt (A.M.L.); pjesteves@cibio.up.pt (P.J.E.); 2 Departamento de Biologia, Faculdade de Ciências da Universidade do Porto, 4169-007 Porto, Portugal; 3 Instituto de Ciências Biomédicas Abel Salazar/Unidade Multidisciplinar de Investigação Biomédica, Universidade do Porto, 4050-313 Porto, Portugal; 4 Unité de Virologie, Immunologie, Parasitologie, Aviaires et Cunicoles, Laboratoire de Ploufragan-Plouzané-Niort, Agence nationale de sécurité sanitaire, de l’alimentation, de l’environnement et du travail (Anses), 22440 Ploufragan, France; evelyne.lemaitre@anses.fr (E.L.); clement.droillard@anses.fr (C.D.); 5 Department of Microbiology, National Veterinary Institute (SVA), Ulls väg 2B, SE75189 Uppsala, Sweden; harri.ahola@sva.se (H.A.); fereshteh.banihashem@sva.se (F.B.); 6 Unité Petite Faune Sédentaire et Espèces Outre-Mer, Direction de la Recherche et de l’Appui Scientifique, Office Français de la Biodiversité (OFB), 44300 Nantes, France; stephane.marchandeau@ofb.gouv.fr; 7 Department of Pathology and Wildlife Diseases, National Veterinary Institute (SVA), Ulls väg 2B, SE75189 Uppsala, Sweden

**Keywords:** recombination, RHDV, GI.1d, GI.3

## Abstract

Recombination is one of the major sources of genetic variation in viruses. RNA viruses, such as rabbit hemorrhagic disease virus (RHDV), are among the viruses with the highest recombination rates. Several recombination events have been described for RHDV, mostly as a consequence of their genomic architecture. Here, we undertook phylogenetic and recombination analyses of French and Swedish RHDV strains from 1994 to 2016 and uncovered a new intergenotypic recombination event. This event occurred in the late 1990s/early 2000s and involved nonpathogenic GI.3 strains as donors for the nonstructural part of the genome of these recombinants, while pathogenic GI.1d strains contributed to the structural part. These GI.3P–GI.1d recombinant strains did not entirely replace GI.1d (nonrecombinant) strains, but became the dominant strains in France and Sweden, likely due to a fitness advantage associated with this genomic architecture. GI.3P–GI.1d (P stands for polymerase) strains persisted until 2013 and 2016 in Sweden and France, respectively, and cocirculated with the new genotype GI.2 in France. Since strains from the first GI.2 outbreaks were GI.3P–GI.2, we hypothesize that GI.3P–GI.1d could be the parental strain. Our results confirm the outstanding recombination ability of RHDV and its importance in the evolution of lagoviruses, which was only revealed by studying complete genomic sequences.

## 1. Introduction

Viruses evolve at a rapid pace, and two mechanisms contribute to their genetic variation: mutation and recombination. In a dynamic interplay, point mutations are an initial source of diversity that might then be shuffled by recombination [1]. Recombination has been recognized as a major force driving RNA-viruses evolution, often associated with host range expansion, increases in virulence, modification of tissue tropism, or emergence of disease outbreaks [2,3,4,5]. Although difficult to establish a cause–effect relationship, there are several pieces of evidence linking recombination to viral emergence in new hosts, including viruses from families *Retroviridae*, *Coronaviridae*, and *Togaviridae* [5]. Recombination happens upon the exchange of genetic material between two related viruses that coinfect the same host cell. As a consequence, RNA viruses experience variable rates of recombination due to the influence of distinct factors, from mechanistic limitations to genomic (in)compatibilities, superinfection exclusion, and interactions between hosts [5,6]. 

As a positive-sense, single-stranded RNA virus, rabbit hemorrhagic disease virus (RHDV) is among the viruses more prone to recombination [5]. This calicivirus (genus *Lagovirus*, family *Caliciviridae*) is the etiological agent of rabbit hemorrhagic disease (RHD), a severely fatal disease for the European rabbit (*Oryctolagus cuniculus*). The first outbreaks of the disease were reported in 1984 in China [7], and later in several countries worldwide (reviewed in Reference [8]). These first strains correspond to variant GI.1c (see nomenclature in Reference [9]), and other variants were later recognized: GI.1a, found mainly in domestic rabbits [10,11]; GI.1b, which was almost entirely restricted to the Iberian Peninsula [12,13]; and GI.1d, occurring in several European countries [14,15]. Less-pathogenic (MRCV) [16] and nonpathogenic strains (GI.3 and GI.4) [17,18,19] have also been recovered. In 2010, a new genotype, GI.2 [20], emerged in France and quickly spread worldwide, causing a massive decline in RHDV-immunized and -naïve domestic and wild populations of European rabbits [21,22,23]. The mechanisms of emergence and evolution of lagoviruses have been studied [24,25,26,27]. The presence of anti-RHDV antibodies in the serum of European rabbits prior to the first outbreaks and the existence of several nonpathogenic lagoviruses point to an evolution from pre-existing nonpathogenic viruses, but a species jump from another leporid species is also a possible scenario [28]. These two hypotheses are not mutually exclusive and both can accommodate recombination as a driver of RHDV evolution, either by generating more virulent genomic combinations or by expanding its host range.

RHDV has a typical nonenveloped virion and a genome of about 7.4 kb, with a viral genome-linked protein (VPg) covalently linked to the 5′ end and a poly-A tail at the 3′ end (reviewed in Reference [8]). The genome has two slightly overlapping open reading frames (ORFs). ORF1 encodes a large polyprotein that, via post-translational proteolytic mechanisms, is cleaved into the mature nonstructural proteins p16, p23, 2C-like helicase (p37), p29, VPg (p13), 3C-like protease (p15), and RNA-dependent RNA polymerase (RdRp), and the major structural capsid protein, VP60. ORF2 encodes for the minor structural protein VP10 [29,30]. RHDV virions also include a subgenomic RNA (sgRNA) of about 2.2 kb, which encodes both the major and the minor structural proteins, and is the main source of structural proteins in infected cells [31,32]. This modular architecture seems to contribute to the existence of a hotspot of recombination at the junction between the nonstructural- and the structural-encoding regions of the genome, as described for other caliciviruses [33,34,35]. Several instances of intergenotypic recombination have been described for RHDV, especially for GI.2, and these almost always involve the combination of pathogenic or nonpathogenic nonstructural proteins with pathogenic structural proteins. Examples include GI.1aP–GI.2 (P stands for polymerase) [36], GI.1bP–GI.2, GI.4P–GI.2 [37], GI.4P–GI.1a [38], and GI.unknownP–GI.1b [39]. Strikingly, the genomes of the first GI.2 outbreaks revealed an existing recombinant history between GI.3 nonstructural proteins and GI.2 capsid proteins [40]. Other instances of recombination events with atypical recombination breakpoints have also been identified [41,42,43,44,45].

Here, we performed a retrospective analysis of rabbit samples collected between 1994 and 2016 in France and Sweden and identified a new recombinant strain, GI.3P–GI.1d, involving nonpathogenic and pathogenic parental donor strains. The recombination event occurred in the late 1990s/early 2000s and the resulting strain successfully persisted in the populations at least until 2016 in France. Its persistence might be attributed to a fitness advantage of having nonstructural nonpathogenic proteins and a pathogenic origin for the capsid proteins. Furthermore, recombination seems highly relevant for RHDV success, as this recombinant appears to be the potential parental strain of recombinant GI.3P–GI.2, which was associated with the first reported GI.2 outbreaks [40]. Our results further confirm the importance of studying the full coding sequences and of monitoring wild rabbit populations for RHDV study.

## 2. Materials and Methods 

### 2.1. Samples

No animals were captured, handled, or killed specifically for the purpose of this study. Liver samples were collected throughout France by the SAGIR network (French wildlife health surveillance network) between 1994 and early 2016, from wild rabbits found dead in the field [46] or from rabbitries. The presence of GI.1 lagoviruses in the samples was diagnosed mainly by the veterinary laboratory Inovalys-Angers (Angers, France) using a GI.1 ELISA test [47]. Positive samples were sent to the Anses laboratory for epidemiological surveillance of French lagoviruses.

The National Veterinary Institute of Sweden (SVA), has a long-standing wildlife disease surveillance program which archives tissues and ancillary data of sick and fallen wildlife. A retrospective search of the database was conducted to identify all cases of rabbit hemorrhagic disease prior to 2013, when GI.2 was first detected in the country [48]. Archived liver tissue samples from six rabbits that died between 1995 and 2012 were included in this study.

### 2.2. Nucleic Acid Extraction, Genome Amplification, and Sequencing

For the French rabbit samples, different methods were used depending on the collection year. An immunocapture RT-PCR test was used to amplify a 501 bp fragment of the 3′ end of the capsid protein VP60 gene [14], or total RNAs were extracted from 100 µL of liver exudate using the RNeasy Mini kit (Qiagen, Hilden, Germany) or the NucleoSpin^®^ RNA kit (Macherey-Nagel, Hoerdt, France). RNAs were reverse-transcribed using oligo(dT) (Invitrogen, Carlsbad, CA, USA) as primer. The cDNAs were then amplified using the PCR primers p33 and p34 (PCR A) [49], RHDVAU and RHDVAL [14], or 14U1 and RVP60-L1 [50], which amplify 501, 561, and 794 bp located at the 3′ end of the gene encoding VP60, respectively. Positive PCR products were purified and quantified. DNA sequences were determined in both directions using the dye terminator method (Life Technologies, Carlsbad, CA, USA) using the PCR primers and analyzed using an automatic DNA sequencer (ABI Prism 3100 Genetic or a 3500 Series Genetic Analyzers, Applied Biosystems, Foster City, CA, USA). To genotype the viruses, the consensus sequences were aligned against nucleotide sequences available in databases using the standard nucleotide BLAST (blastn; NCBI web BLAST service). Complete coding sequences of six selected French GI.1d viruses were obtained by overlapping PCRs that generated fragments between 2500 and 7300 bp in length (primer sequences available upon request). At least one PCR covered the major recombinant breakpoint located between the nonstructural- and structural-encoding genes of lagoviruses [37,41]. PCR products were purified and sequenced as described above using the PCR primers and several inner primers (primer sequences available upon request). The 5′ and 3′ genome regions were obtained for five out of the six GI.1d viruses using the rapid amplification of cDNA ends (RACE) method following the protocol developed in Reference [51]. PCR products were sequenced as described above and sequences were compiled using Vector NTI Advance 11.5 software (Life Technologies, Carlsbad, CA, USA). To further determine whether other GI.1d strains collected between 1994 and early 2016 in France were recombinants, a 518 bp long region located in the 5′ end of the nonstructural part of the genome encompassing proteins p16 and p23 (nt 13 to 530) was amplified using primers 1U and 1L (PCR 1) and sequenced as described elsewhere [52].

For the Swedish rabbit samples, these were disrupted and homogenized in 750 μL of lysis buffer with 400 mg of 2.0 mm zirconia beads (BioSpec Products Inc., Bartlesville, OK, USA), using a FastPrep homogenizer (MP Bio, Santa Ana, CA, USA). Each sample was homogenized two times in a 3 min run at 6.5 m/s and centrifuged at 15,000× *g* in 4 °C for 10 min. Supernatants were used for nucleic acid extraction. Total nucleic acids were extracted using the Viral NA Magnetic Beads kits in an ArrowTM 2 extraction robot (DiaSorin, Saluggia, Italy), following manufacturer’s instructions. Sequencing libraries were constructed using the Nextera XT DNA Library Preparation Kit (Illumina Inc., San Diego, CA, USA) according to the manufacturer’s instructions. Sequencing of each library was performed on a MiSeq Instrument (Illumina Inc., San Diego, CA, USA), using a Miseq Reagent Kit v3 in a 600 cycle paired-end run. Quality analysis, filtering, and downstream sequence analysis of the raw reads were performed using CLC genomics workbench 10.0.1 (CLC bio, Aarhus, Denmark).

### 2.3. Recombination Analysis 

The complete coding sequences obtained in this study were aligned in BioEdit software version 7.0.3 [53] with publicly available complete coding sequences of lagoviruses representing genotypes GI.1, GI.2, GI.3, and GI.4. The final dataset included 237 sequences, 7369 nucleotides in length. The dataset was screened for recombination with RDP software version 4.40 [54], with the following parameters: sequences were set to linear, Bonferroni correction, highest acceptable *p*-value of 0.05, and 100 permutations. Only recombination events detected by three or more methods were considered.

### 2.4. Phylogenetic Analyses

Maximum-likelihood (ML) phylogenetic trees were inferred in MEGA6 [55] for the 237 sequences. A ML tree was inferred for the capsid VP60 gene for genotype and variant assignment of the sequences obtained in the study. A ML tree was further inferred for the nonstructural part of the genome, according to the recombination breakpoint identified by RDP. The best model of nucleotide substitution was determined for each ML tree in MEGA6 as the model with the lowest AICc value (Akaike information criterion, corrected). Support for each cluster was obtained from 1000 bootstrap replicates.

ML trees were also estimated as described above for p16/p23 and partial VP60 gene for 67 French and 6 Swedish GI.1d strains (including the strains for which the entire coding genomic sequences were obtained in this study; see Supplementary Information). Sequences from a few reference GI.1 variants and all GI.1d available in public databases, as well as six French GI.1a and GI.1b strains, the GI.3 strain 06-11 (MN737115), the GI.4 strain MIC-07 (EU871528), and MRCV (GQ166866) were included.

## 3. Results and Discussion

In lagoviruses, a recombination hotspot at the RdRp/VP60 transition, corresponding in the genome to the nonstructural/structural protein boundary, has been consistently identified [36,37,38,39,42]. Recombination shapes RNA viruses’ genetics and evolution, and its evolutionary significance might have been overlooked in the RHDV strains that have circulated worldwide since late 1980s [8]. To fill this gap, we performed a retrospective analysis of RHDV strains isolated from wild rabbits collected in France and Sweden from the late 1990s to mid-2010s, spanning more than 20 years of evolution.

We obtained 12 new full coding sequences of lagoviruses from 1995–2016: six were collected in Sweden and six collected in France (Table 1). Genomic sequences were deposited in GenBank under the accession numbers MH190418 (French strains 00-21), MT628287–MT628291 (French strains 95-10, 05-01, 09-02, 09-03, and 16-09, respectively), and MT819374–MT819379 (Swedish strains 96-VLT000113, 03-VLT001218, 06-VLT001843, 10-VLT001467, 12-VLT000099, and 12-VLT000101, respectively). Based on the capsid sequence, they were assigned as GI.1d (data not shown) [9]. No new insertions or deletions were observed in the coding sequences, and a Blast search of their full coding sequences revealed 89.83%–98.57% nucleotide identities to European strains ZD0 (GI.1d), BLA_1994 (GI.1d), BS89 (GI.1d), Zar11-11 (GI.2), and Zar06-12 (GI.2) (accession numbers KU882095, KP144792, X87607, KP129398, and KP129399, respectively). For an additional 61 French samples, partial sequences were also obtained for p16 and p23 (nucleotides 19–484) and for VP60 (with the exception of samples 94-02, 00-13 and 03-24, for which VP60 coding sequences were already available: accession numbers AJ535102, AJ495856, and AJ969628, respectively). These 61 samples span 22 years of RHDV circulation in France (1994–2016). These additional sequences were submitted to the European Nucleotide Archive (ENA) and have the following accession numbers: LR862100–10, LR862154–99, LR862291–92; LR862294-352 and LR877009-10.

Full coding sequences of RHDV (genotypes GI.1–4) were retrieved from public databases. The final dataset consisted of 237 sequences, 7369 nucleotides in length. Sequences were screened for recombination using RDP [54]. A recombination breakpoint was detected, with strong statistical support (*p*-values < 0.05; Table 2), at position 5426 (5384–5444; 99% confidence interval) using six methods implemented in RDP (RDP, BootScan, MaxChi, Chimaera, SiScan, and 3Seq). This recombination breakpoint was identified for eight strains, five from Sweden and three from France: 03-VLT001218, 06-VLT001843, 10-VLT001467, 12-VLT000099, 12-VLT000101, 05-01, 09-03, and 16-09. RDP detected 06-11 (nonpathogenic strain; France 2006; genotype GI.3) as the most likely donor strain for the fragment corresponding mostly to the nonstructural part, and 00-21 (pathogenic strain; France 2000; genotype GI.1d) as the most likely donor strain for the downstream fragment. No recombination breakpoints were detected for strains 96-VLT000113, 95-10, 00-21, or 09-02.

**Figure 1:**
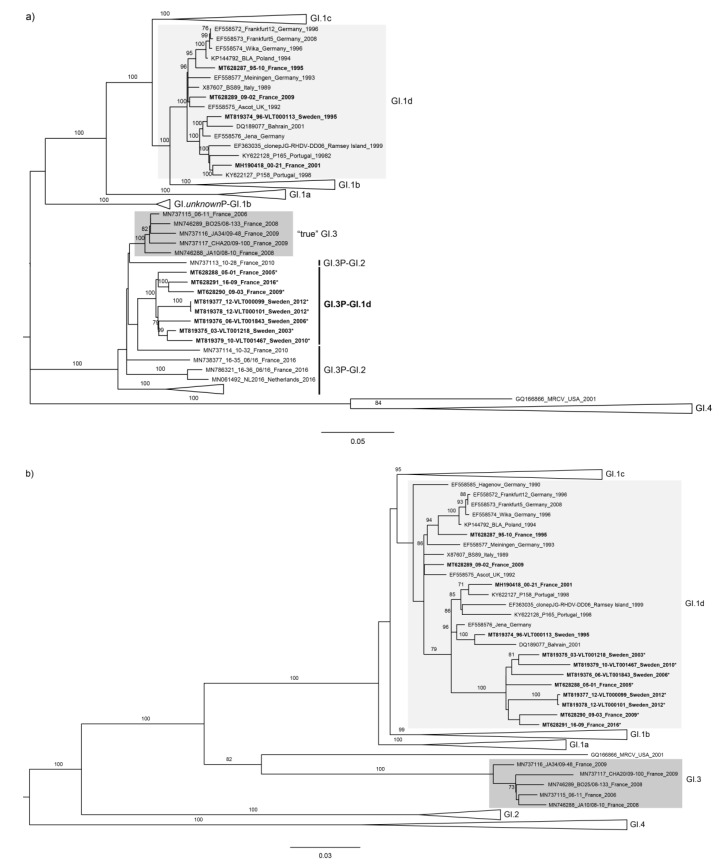
Maximum likelihood (ML) phylogenetic trees for (**a**) the nonstructural genes (*n* = 237 sequences; nucleotides 1–5295; nucleotide substitution model GTR+G+Γ4) and (**b**) the structural genes (*n* = 237 sequences; nucleotides 5296–7369; nucleotide substitution model GTR+G+Γ4). Horizontal branch lengths are drawn to scale of nucleotide substitutions per site, and the trees are midpoint-rooted. The percentage of trees in which the associated taxa clustered together was determined from 1000 bootstrap replicates and is shown next to the branches (only bootstrap values ≥ 70 are shown). Sequences obtained in this study are shown in bold. Recombinant strains are marked with *. GenBank accession numbers of the sequences that appear in the collapsed branches are listed in the supplementary information (Supplementary Table S1).

In accordance with the RDP results, maximum likelihood (ML) trees were built for two fragments independently, splitting the coding sequences into nonstructural and structural proteins (Fragment 1: nucleotides 1–5426; Fragment 2: nucleotides 5427–7369). For Fragment 1, strains 96-VLT000113, 95-10, 00-21, and 09-02 clustered with other GI.1d sequences (bootstrap value of 100). In contrast, strains 03-VLT001218, 06-VLT001843, 10-VLT001467, 12-VLT000099, 12-VLT000101, 05-01, 09-03, and 16-09 clustered together in a highly supported group (bootstrap value of 100; Figure 1a). Importantly, they clustered with GI.3 strains (e.g., 06-11, GenBank accession number FR851337), as well as with the newly identified GI.3P–GI.2 recombinants (e.g., Zar11-11, GenBank accession number KP129398; bootstrap value of 100) [40]. For Fragment 2 (VP60 and VP10), the strains obtained in this study all clustered with GI.1d sequences (Figure 1b). Although this cluster was not well resolved (bootstrap value < 70), they were included in the broader group of GI.1a–d, which was well supported (bootstrap value 100). Therefore, the phylogenetic analysis further confirmed a recombination breakpoint at the RdRp/VP60 boundary for the eight sequences identified by RDP (c.f. Table 2).

RHDV was first reported in 1988 in France [56] and in 1990 in Sweden [57], and became endemic. In France, phylogenetic analysis showed that GI.1b and GI.1c cocirculated at the time of emergence, but these strains were then progressively replaced by GI.1d in the 1990s [14,49]. In Sweden, information is rather scarce, but earlier strains were GI.1c (Sweden 90, GenBank accession number U65350) and GI.1d (Sweden 94, GenBank accession number U65351), and a replacement pattern is unknown [58]. Our results identified a new recombinant strain, GI.3P–GI.1d, which links nonpathogenic nonstructural elements with a pathogenic capsid. The ML trees for the partial p16+p23 and VP60 (Supplementary Figure S1) showed that GI.3P–GI.1d emerged as early as 2000 in France and 2003 in Sweden and was able to spread in French (Supplementary Figure S2) and Swedish rabbit populations, and persisted for more than 10 years. In fact, from our 73 sequences (6 full coding sequences from Sweden, 61 partial and 6 full coding sequences from France), 48 were GI.3P–GI.1d recombinants (65.8%). Considering only sequences collected from 2000 to 2016 (*n* = 66), 72.7% were GI.3P–GI.1d recombinants. The remaining 25 of 73 sequences were assigned to nonrecombinant GI.1d group (34.2%). When looking to the relative annual frequency of GI.3P–GI.1d vs. “true” GI.1d, it was always higher for GI.3P–GI.1d from 2001 onwards with only two exceptions: 2010 and 2011. Within a few years, the combination of nonpathogenic and pathogenic viruses had almost replaced the latter. After GI.2 emerged in France and Sweden in 2010 and 2013, respectively, GI.3P–GI.1d strains started to disappear from the populations (only sequences 13-01, 14-14, and 16-09 were recovered after 2012 in France) until 2016, after which they were not further identified (Le Gall-Reculé, unpublished observations). Since the strains associated with GI.2 emergence in France in 2010 are GI.3P–GI.2 [20,40] and GI.3P–GI.1d were the dominant strains, we hypothesize that GI.3P–GI.1d might have been the donor of the nonstructural proteins of GI.3P–GI.2, although we cannot fully confirm this observation. Interestingly, the GI.3P–GI.2 strains again combine a nonpathogenic nonstructural donor with a potential pathogenic structural donor. It remains to be tested whether the unique features of GI.2 are conferred by this genomic architecture [20,50,59,60].

While GI.3P–GI.1d did not completely replace GI.1d, it might present a selective advantage over nonrecombinant GI.1d. Indeed, no obvious differences in mortality rates were noticed in the field that could indicate putative shifts in virulence, but other viral features might have been altered that promoted a higher fitness, such as in virus replication or transmission effectiveness, which allowed GI.3P–GI.1d to remain in the population. A scenario of a selective advantage of recombinant over parental viral strains has been hypothesized for the closely related noroviruses, where transmission effectiveness surpasses putative replicative disadvantages [61]. In the absence of such a selective advantage, GI.3P–GI.1d strains would eventually become extinct and disappear from the population, as previously observed for the GI.unknownP–GI.1b strain identified in the 1990s in Portugal [39]. Instead, the new recombinant successfully circulated in rabbit populations in at least two countries, France and Sweden, and was consistently detected in both, unlike the original GI.1d. However, we cannot discard the possibility that the increase in frequency of GI.3P–GI.1d was due to genetic drift following the emergence of this recombinant.

As for emergence of GI.3P–GI.1d, we hypothesize that it occurred through a single event of recombination rather than via independent events in each country. Given the distance and geographic barriers between France and Sweden, it is likely that anthropogenic factors were at the origin of GI.3P–GI.1d arrival to the second country. The detection of this recombinant first in France in 2000 and only three years later in Sweden suggests that it originated in France. Nonetheless, the phylogenetic analysis did not provide enough resolution to test this hypothesis and the lack of a systematic sampling, in particular in Sweden, did not allow the country of origin to be entirely confirmed.

Recombination at the RdRp/VP60 boundary is facilitated by a weak interaction between the RNA template and the viral polymerase [62] which, due to complex secondary structures, dissociates from the original strand carrying its RNA strand. The high nucleotide identity in this region and the presence of the sgRNA from another virion, the 5′ end of which is collinear with this region and acts as an acceptor molecule, assists the switch to a new strand [5]. This homologous copy-choice model of recombination at the RdRp/VP60 boundary is the most likely to occur in lagoviruses. The lack of full genomic sequences from the first RHDV outbreaks hampered the identification and the true assessment of the frequency of recombination in RHDV, but recombination has clearly assisted RHDV evolution, as demonstrated by the identification of a Mexican strain from 1989 as an intravariant recombinant with a breakpoint close to the RdRp/VP60 boundary [41]. Thus, intravariant recombination also occurs in RHDV, certainly undergoing unnoticed due to high sequence homology. As suggested for noroviruses [61], intravariant recombination might indeed be more frequent than intervariant or intergenotype recombination, but only highly sensitive methods and close monitoring allows their detection.

## 4. Conclusions

Our results confirm the importance of recombination in generating diversity in lagoviruses, as reported previously (e.g., [37,39,41,63]), which may act as a survival strategy for these viruses. Indeed, there is growing evidence for recombination in lagoviruses, especially at the start of the structural genes, an hotspot of recombination extensively documented in caliciviruses [33,34,35,64]. Maintenance of recombinant strains combining nonpathogenic nonstructural sequences with pathogenic structural sequences, with the disappearance of “true” pathogenic genomes, suggests that the nonpathogenic nonstructural sequences can confer additional epidemiological benefits over pathogenic nonstructural sequences. Recombination clearly contributes to the complexity of RHDV evolution. For the closely related norovirus, the impact of recombination in shaping genotype evolution was only uncovered when more than just ORF1 was sequenced [33]. Hence, the analysis of full genomes is of utmost importance in studying the evolution of lagoviruses. Furthermore, new strains with distinct features and levels of pathogenicity seem to quickly arise in the wild due to recombination, which reinforces the role of wildlife monitoring in documenting interactions between RHDV genotypes.

## Figures and Tables

**Table 1:** Collection date and location of full genome sequences obtained in this study.

Sample Code	Location (Department or Municipality, Country)	Collection Date (Month-Year)	Accession Number
95-10	Marne, France	05-1995	MT628287
00-21	Aude, France	08-2000	MH190418
05-01	Manche, France	01-2005	MT628288
09-02	Aveyron, France	01-2009	MT628289
09-03	Bouches du Rhône, France	02-2009	MT628290
16-09	Loire, France	01-2016	MT628291
96-VLT000113	Gotland, Sweden	12-1995	MT819374
03-VLT001218	Rönneby, Sweden	11-2003	MT819375
06-VLT001843	Kävlinge, Sweden	09-2006	MT819376
10-VLT001467	Gotland, Sweden	08-2010	MT819379
12-VLT000099	Tomelilla, Sweden	01-2012	MT819377
12-VLT000101	Tomelilla, Sweden	01-2012	MT819378

**Table 2:** Recombination results from the RDP software analysis.

		Most Likely Donor Strain		Methods and Average *p*-Values					
Strains	Recombination Breakpoint (Nucleotide Positions) ^1^	Nonstructural Proteins	Structural Proteins	RDP	BootScan	MaxChi	Chimaera	SiScan	3Seq
05-01	5426 (5384–5444)	06-11 (GI.3)	00-21 (GI.1d)	2.024 × 10^−12^	6.164 × 10^−11^	6.146 × 10^−19^	2.023 × 10^−19^	7.051 × 10^−38^	3.270 × 10^−62^
09-03									
16-09									
03-VLT001218									
06-VLT001843									
12-VLT000099									
12-VLT000101									
10-VLT001467									

^1^ 99% confidence interval.

## Supplementary Materials

The following are available online at https://www.mdpi.com/2073-4425/11/8/910/s1, Figure S1: Maximum likelihood (ML) phylogenetic trees for (a) partial p16+p23 (*n* = 97 sequences; nucleotides 19–484; nucleotide substitution model GTR) and (b) partial VP60 (*n* = 97 sequences; nucleotide substitution model GTR). All available GI.1d sequences were included in the analysis. Horizontal branch lengths are drawn to scale of nucleotide substitutions per site. The sequence of the European brown hare syndrome virus (EBHSV strain GD, Z69620) was used as an outgroup to root the trees. Reliability of the trees was assessed by bootstrap with 1000 replicates and is shown next to the branches (only bootstrap values ≥ 70 are shown). Sequences underlined are the oldest in groups GI.3 and GI.1d and sequences collected after 2000 appear in bold. GenBank and European Nucleotide Archive (ENA) accession numbers are provided. Figure S2: Spatial distribution of 57 GI.1d and GI.3P–GI.1d outbreaks in rabbit populations in France between 1994 and 2016. Data are pooled by department (administrative area) and the color indicates the lagoviruses involved in the outbreaks. For 4 strains, no information on the location was available. Table S1: List of the 237 sequences used in this study and the genotype/variant they belong to according to their VP60.
